# Prediction of pathologic complete response prediction in patients with locally advanced esophageal squamous cell carcinoma treated with neoadjuvant immunochemotherapy: A real-world study

**DOI:** 10.17305/bjbms.2022.7696

**Published:** 2023-01-06

**Authors:** Jifeng Feng, Liang Wang, Xun Yang, Qixun Chen, Xiangdong Cheng

**Affiliations:** 1 The Second Clinical Medical College, Zhejiang Chinese Medical University, Hangzhou, Zhejiang, China; 2 Department of Thoracic Oncological Surgery, Cancer Hospital of University of Chinese Academy of Sciences, Zhejiang Cancer Hospital, Hangzhou, China; 3 Zhejiang Provincial Research Center for Upper Gastrointestinal Tract Cancer, Cancer Hospital of University of Chinese Academy of Sciences, Zhejiang Cancer Hospital, Hangzhou, China

**Keywords:** Esophageal squamous cell carcinoma (ESCC), systemic immune-inflammation index (SII), neutrophil lymphocyte ratio (NLR), prognostic nutritional index (PNI), lymphocyte monocyte ratio (LMR), platelet lymphocyte ratio (PLR), pathologic complete response (pCR)

## Abstract

As an emerging hotspot for patients with locally advanced esophageal squamous cell carcinoma (LA-ESCC), neoadjuvant immunochemotherapy (nICT) is safe and feasible. Pathological complete response (pCR) is considered to be an important therapeutic effect of neoadjuvant therapy. However, few studies have explored pCR predictors for nICT in LA-ESCC. The purpose of this study was to predict pCR after nICT in LA-ESCC by pretreatment clinical characteristics and hematological indexes. The primary endpoint was to explore the impacts on the predictors for pCR prediction. Clinical characteristics and hematological indexes, including systemic immune-inflammation index (SII), neutrophil lymphocyte ratio (NLR), lymphocyte monocyte ratio (LMR), prognostic nutritional index (PNI), and platelet lymphocyte ratio (PLR), were conducted. A total of 150 LA-ESCC patients were enrolled in the current study. There were 14 (9.3%) female and 136 (90.7%) male patients. Fifty-two patients achieved pCR (34.7%). A higher pCR rate was found in low-NLR group (43.7% vs. 26.6%, *P* = 0.028) and high-LMR group (43.8% vs. 21.3%, *P* = 0.004), respectively. Differentiation [odds ratio (OR) = 0.464, 95% confidence interval (CI) = 0.259–0.830, *P* = 0.010], LMR (OR = 0.309, 95% CI = 0.132–0.707, *P* = 0.007), and clinical TNM (cTNM) (OR = 0.225, 95% CI = 0.115–0.441, *P* < 0.001) were the independent predictors for pCR. The nomogram for pCR prediction based on LMR, differentiation, and cTNM stage had good discrimination performance and calibration coordination (C-index = 0.779). The results of our study are of great significance for designing therapeutic strategies. Nomogram based on LMR, differentiation, and cTNM may accurately and effectively predict pCR.

## Introduction

There were about 0.60 million new cases and 0.54 million deaths of esophageal cancer (EC) worldwide in 2020 [1]. EC was the sixth (320,000 new cases) leading cancer type and the fourth (300,000 deaths) most common cause of death in China in 2020 [2]. Therefore, more than half of the new cases and deaths of EC [mainly esophageal squamous cell carcinoma (ESCC), which accounts for more than 90%] occurred in China. Neoadjuvant chemotherapy (nCT) or chemoradiotherapy (nCRT) followed by surgery is the preferred therapy for locally advanced ESCC (LA-ESCC) [3, 4]. However, the long-time survival for nCT or nCRT plus surgery in LA-ESCC is still unsatisfactory [5]. Recently, immunochemotherapy (ICT) has become one of the important regimens and has achieved remarkable results in advanced ESCC [6, 7]. Following encouraging results in advanced ESCC, neoadjuvant ICT (nICT) has gained much attention. Clinical evidence reveal that nICT in LA-ESCC is safe and feasible [8–12].

**Figure 1.**
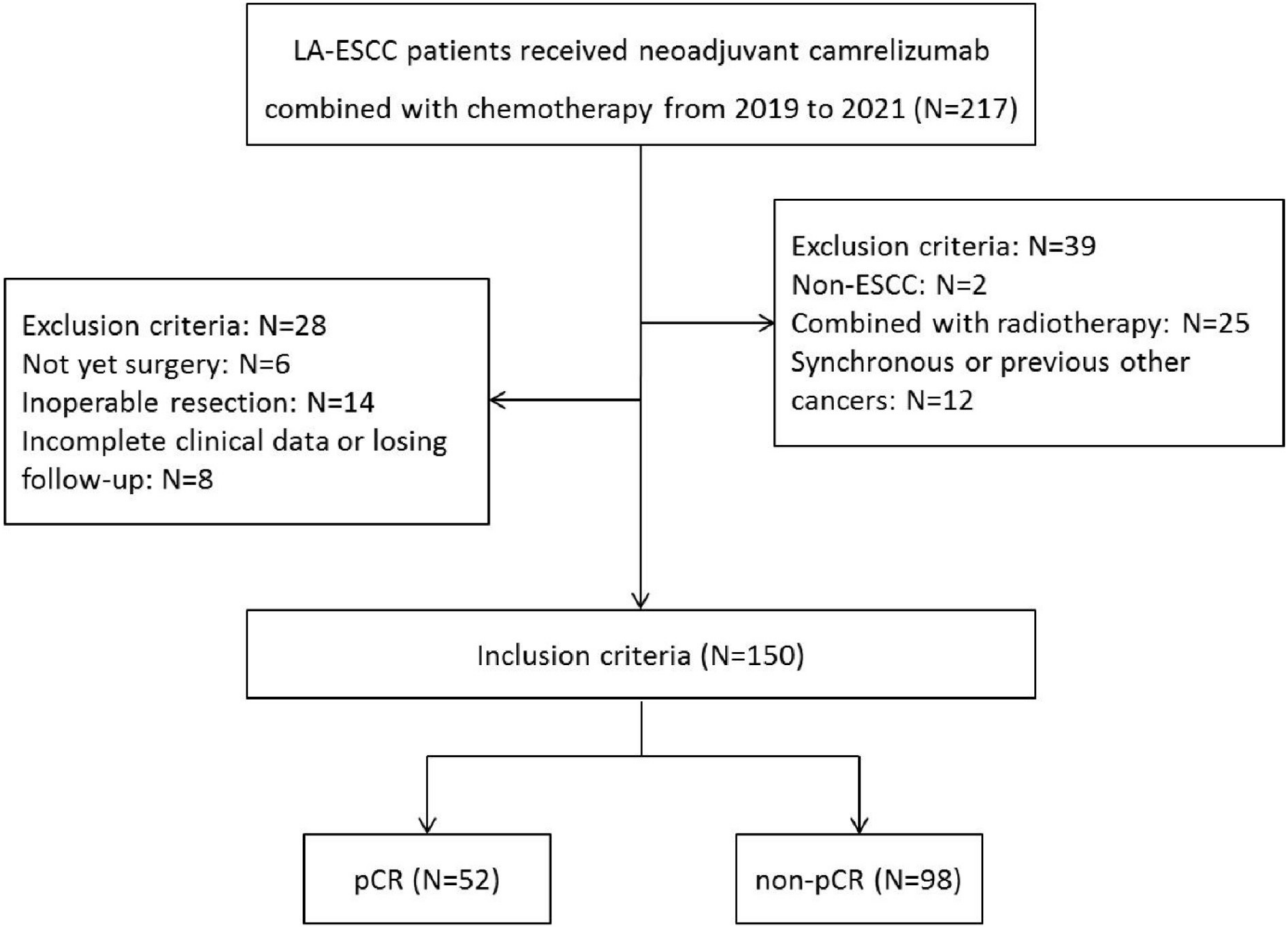
**The flow diagram of selection of eligible LA-ESCC patients who received nICT followed by radical resection.** Based on the inclusion and exclusion criteria, a total of 150 patients were enrolled. LA-ESCC: locally advanced esophageal squamous cell carcinoma; nICT: neoadjuvant immunochemotherapy; pCR: Pathological complete response.

Pathological complete response (pCR) is considered to be an important therapeutic effect of neoadjuvant therapy [13]. However, no reliable indicators can be used to predict pCR for neoadjuvant therapy in ESCC. Hematological indexes, such as neutrophil to lymphocyte ratio (NLR), systemic immune-inflammation index (SII), platelet to lymphocyte ratio (PLR), prognostic nutritional index (PNI), and lymphocyte to monocyte ratio (LMR), were related to pCR and prognosis in several cancers after neoadjuvant treatment [14–17]. As an emerging treatment pattern in LA-ESCC with nICT, however, there are few studies regarding predictors for pCR prediction. Recently, a study including 64 cases of ESCC explored the associations between hematological indexes and pCR in nICT [18]. Another study including 79 cases of ESCC established a nomogram to predict tumor regression grade (TRG), but not for pCR, in nICT [19]. However, the sample sizes of these two studies were small. Moreover, the predictors for pCR in nICT are still unclear in LA-ESCC patients.

The knowledge of predictors for pCR will be of great value in designing treatment strategies. Therefore, we aimed to predict pCR in nICT with pretreatment clinical characteristics and hematological indexes in LA-ESCC. Moreover, a nomogram model in LA-ESCC was also developed to predict pCR in nICT.

## Materials and methods

### Study design and patient selection

The study enrolled LA-ESCC patients receiving nICT (camrelizumab plus chemotherapy) followed by surgery in the period between June 2019 and December 2021. The inclusion criteria for the retrospective study involved the following: (1) ESCC confirmed by histopathology; (2) LA-ESCC with clinical TNM (cTNM) stage II-IVA; (3) neoadjuvant camrelizumab combined with chemotherapy; (4) radical resection (R0) after nICT; and (5) complete medical records. Patients with any infectious, autoimmune, or hematologic disease were excluded. Patients with other previous or synchronous cancers were also excluded. The detailed criteria are shown in Figure 1. Finally, 150 LA-ESCC patients were enrolled in the current study.

### Treatment strategies

Prior to surgery, all patients in the current study received two cycles of nICT. Carboplatin (5 mg/ml per minute for the area under the curve) on day 1, albumin-bound paclitaxel (100 mg/m^2^) on days 1 and 8, and camrelizumab (200 mg) on day 1 were administered intravenously every three weeks. Clinical effect evaluation was performed after two therapy cycles. The open or laparo-thoracoscopic McKeown or Ivor Lewis procedures with twofield lymphadenectomy were performed within 4–6 weeks after the completion of nICT [20, 21]. The clinical TNM stage was determined according to the eighth AJCC/UICC TNM staging system [22]. The pCR was defined as no evidence of residual tumor cells (ypT0N0M0) [23].

### Hematological indexes definition

The medical data were retrospectively collected, such as pretreatment clinical characteristics and hematological indexes from medical records. The blood indexes were obtained within one week before nICT, such as neutrophils, lymphocytes, platelets, monocytes, albumin, and so on. The pretreatment blood indexes were defined as neutrophils divided by lymphocytes (NLR), platelets divided by lymphocytes (PLR), and lymphocytes divided by monocytes (LMR), respectively. The PNI and SII were defined as follows: PNI is measured by preoperative [10×albumin (g/dl)] + [0.005 × lymphocytes (/mm^3^)] [16]. The SII was defined as platelets × neutrophil/lymphocytes [17]. The reportings of clinicopathological variables and hematological indicators were conducted in accordance with the REMARK guidelines [24].

### Ethical statement

This study was conducted in accordance with the Declaration of Helsinki. Informed consent was signed by each patient in the current study. The ethics committee of Zhejiang Cancer Hospital approved the study (IRB-2020-183).

### Statistical analysis

Statistical analyses were performed by R software (version 4.1.2), SPSS 20.0, and Medcalc 17.6. Student’s t-tests (normal distribution) or Mann–Whitney U-tests (non-normal distribution) were used for continuous variables. Chi-square or Fisher’s exact tests were carried out to analyze categorical variables. The optimum cut-off values for PLR, NLR, LMR, SII, and PNI were performed with the cutoff finder [25]. To better understand the predictive ability for pCR, the AUCs were compared by ROC curves. Odds ratios (ORs) and 95% confidence intervals (CIs) in logistic regression analyses were carried out to identify the predictors for pCR. A nomogram was built for pCR prediction and assessed the discrimination performance and calibration coordination. The internal validation was performed by a calibration curve. The decision curve analysis (DCA) and AUC were calculated to quantify the ability of pCR prediction. All tests were two-sided, and *P*-value <0.05 indicated statistical significance.

### Data availability

The datasets used in the study are available from the corresponding author on reasonable request.

## Results

### Patient characteristics

A total of 150 LA-ESCC patients were recruited in this study. There were 14 (9.3%) female and 136 (90.7%) male patients. The mean age was 62.7 ± 6.7 years. Most patients were diagnosed at the stage of cT3 (65.4%), cN1 (59.3%), and cTNM III (54.7%), respectively. Fifty-two patients achieved pCR (34.7%). The detailed baseline characteristics, as well as hematological indicators are shown in Table 1.

**Table 1:** Baseline characteristics for 150 LA-ESCC patients

**Characteristics**	**Value**
Age (mean ± SD, years)	62.7 ± 6.7
Sex (male/female, %)	136 (90.7)/14 (9.3)
ECOG-PS (0/1, %)	115 (76.7)/35 (23.3)
BMI (mean ± SD, Kg/m^2^)	20.16 ± 2.60
Tumor location (upper/middle/lower, %)	13 (8.7)/84 (56.0)/53 (35.3)
Differentiation (well/moderate/poor, %)	23 (15.3)/70 (46.7)/57 (38.0)
Hypertension history (yes/no, %)	41 (27.3)/109 (72.7)
Diabetes history (yes/no, %)	16 (10.7)/134 (89.3)
Smoking history (yes/no, %)	100 (66.7)/50 (33.3)
Drinking history (yes/no, %)	106 (70.7)/44 (29.6)
cT stage (T2/T3/T4a, %)	26 (17.3)/98 (65.4)/26 (17.3)
cN stage (N0/N1/N2/N3, %)	26 (17.3)/89 (59.3)/31 (20.7)/4 (2.7)
cTNM stage (II/III/Iva, %)	42 (28.0)/82 (54.7)/26 (17.3)
pCR (yes/no, %)	52 (34.7)/98 (65.3)
Inflammatory and nutritional indexes	
Neutrophils (mean ± SD, 10ˆ9/L)	4.93 ± 1.71
Lymphocytes (mean ± SD, 10ˆ9/L)	1.60 ± 0.52
Monocytes (mean ± SD, 10ˆ9/L)	0.46 ± 0.16
Platelets (mean ± SD, 10ˆ9/L)	236.9 ± 73.1
Albumin (mean ± SD, g/dL)	4.11 ± 3.43
NLR (mean ± SD)	3.36 ± 1.63
PLR (mean ± SD)	162.9 ± 72.9
LMR (mean ± SD)	3.80 ± 1.55
PNI (mean ± SD)	49.05 ± 4.42
SII (mean ± SD)	828.7 ± 580.1

LA-ESCC: locally advanced esophageal squamous cell carcinoma; SD: standard deviation; ECOG-PS: eastern cooperative oncology group performance status; BMI: body mass index; TNM: tumor node metastasis; pCR: pathological complete response; NLR: neutrophil to lymphocyte ratio; PLR: platelet to lymphocyte ratio; LMR: lymphocyte to monocyte ratio; SII: systemic immune-inflammation index; PNI: prognostic nutritional index.

### Baseline characteristics grouped by pCR

**Figure 2.**
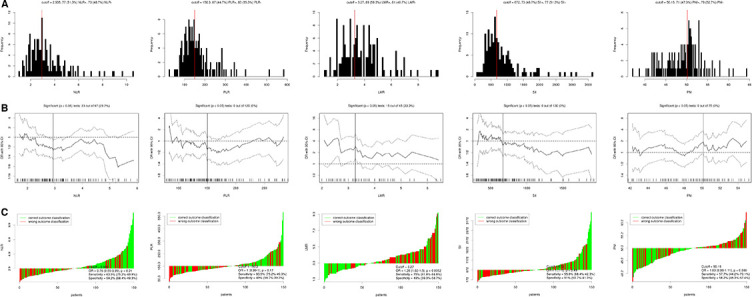
**The optimal cutoff value achieved for hematological indicators.** (A) Distribution for NLR-, PLR-, LMR-, SII-, and PNI-based cutoff optimization. (B) Cutoff optimization for NLR, PLR, LMR, SII, and PNI by correlation with pCR prediction. (C) Waterfall plot regarding cutoff value for NLR, PLR, LMR, SII, and PNI. NLR: neutrophil lymphocyte ratio; PLR: platelet to lymphocyte ratio; LMR: lymphocyte to monocyte ratio; SII: systemic immune-inflammation; PNI: prognostic nutritional index.

**Figure 3.**
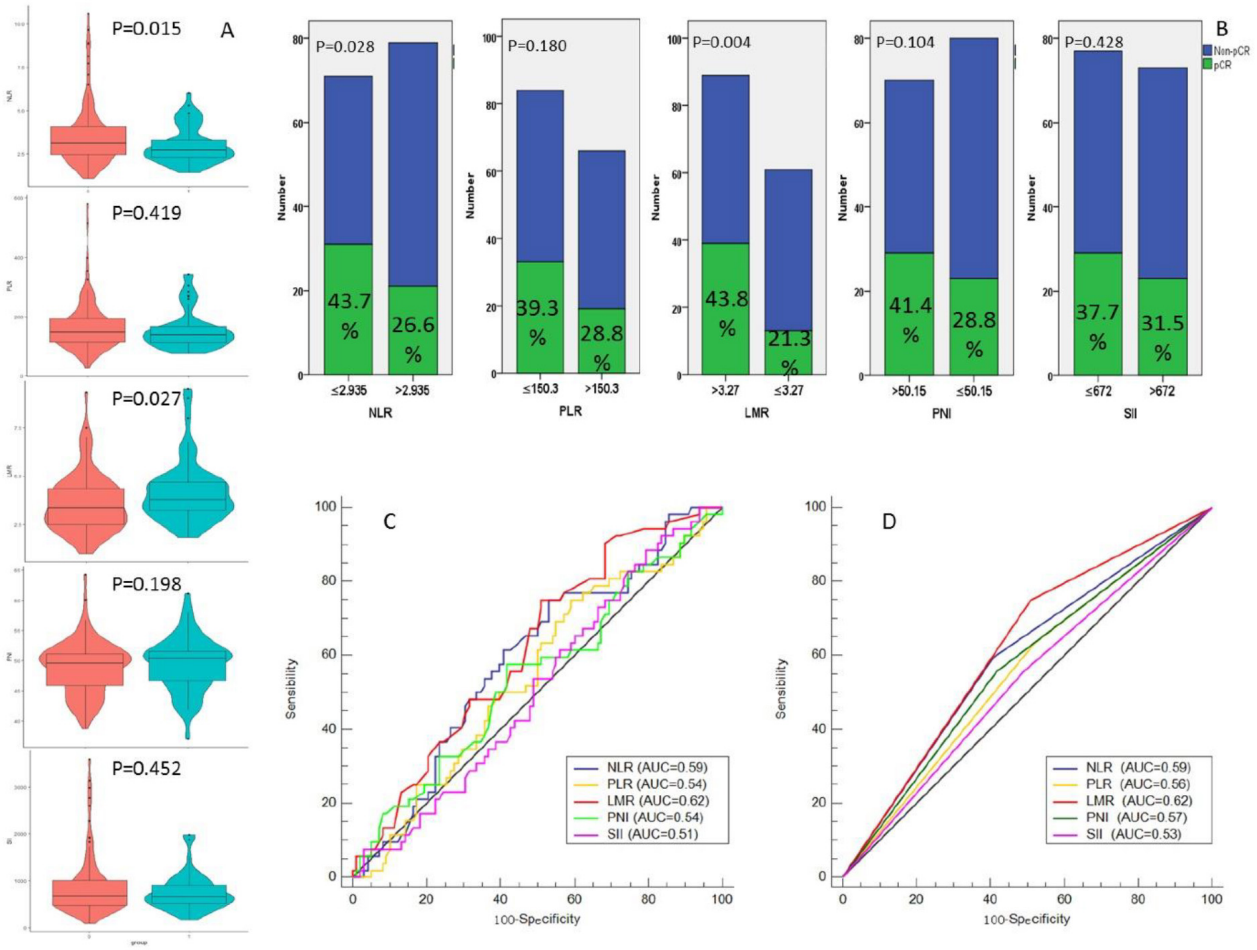
**The violin plots, histograms, and ROC curves.** (A) The violin plots about the values of hematological indicators grouped by pCR. (B) The pCR rates grouped by hematological indexes. (C) ROC curve for pCR prediction based on continuous hematological indicators. (D) ROC curve for pCR prediction based on categorical hematological indicators. pCR: Pathological complete response; NLR: Neutrophil lymphocyte ratio; PLR: Platelet to lymphocyte ratio; LMR: Lymphocyte to monocyte ratio; SII: Systemic immune-inflammation; PNI: Prognostic nutritional index.

According to the cut-off finder, the optimal cut-off levels of PLR, NLR, LMR, PNI, and SII were 150.3, 2.935, 3.27, 50.15, and 672, respectively (Figure 2). The levels of hematological indexes grouped by pCR are shown in Figure 3A. The values of NLR (2.98 ± 1.06 vs. 3.56 ± 1.83, *P* = 0.015) and LMR (4.18 ± 1.60 vs. 3.60 ± 1.49, *P* = 0.027) in patients who achieved pCR were significantly lower and higher than those with non-pCR, respectively. Significantly higher pCR rates were found in low-NLR group (43.7% vs. 26.6%, *P* = 0.028) and high-LMR group (43.8% vs. 21.3%, *P* = 0.004) (Figure 3B). ROC curves for pCR prediction based on hematological indexes are shown in Figure 3C-D. Regarding clinical characteristics, a significantly higher pCR rate was found in well differentiation, early cT stage, early cN stage, and early cTNM stage (Table 2).

### Predictors of pCR with logistic analyses

The results of univariate and multivariate logistic regression analyses are shown in Table 3. Univariate analyses revealed that NLR, LMR, cTNM stage, cT stage, cN stage, and differentiation were the predictors of pCR. Multivariate analyses then reported that patients in high-LMR group (OR = 0.309, 95% CI = 0.132–0.707, *P* = 0.007), well differentiation (OR = 0.464, 95% CI = 0.259–0.830, *P* = 0.010) and early cTNM stage (OR = 0.225, 95% CI = 0.115–0.441, *P* < 0.001) were more inclined to achieve pCR after nICT.

**Table 2:** Comparison of the baseline characteristics in LA-ESCC grouped by pCR

	**pCR (*n* = 52)**	**Non-pCR (*n* = 98)**	***P*-value**
Age (≤60/>60, years)	16 (30.8)/36 (69.2)	38 (38.8)/60 (61.2)	0.331
Sex (female/male)	7 (13.5)/45 (86.5)	7 (7.1)/91 (92.9)	0.205
ECOG-PS (0/1)	40 (76.9)/12 (23.1)	75 (76.5)/23 (23.5)	0.957
BMI (≤20/>20, kg/m^2^)	28 (53.8)/24 (46.2)	47 (48.0)/51 (52.0)	0.493
Tumor location (U/M/L)	4 (7.7)/32(61.5)/16(30.8)	9 (9.2)/52(53.1)/37(37.7)	0.226
Differentiation (W/M/P)	15(28.9)/23(44.2)/14(26.9)	8 (8.2)/47(48.0)/43(43.8)	0.002
Hypertension history (N/Y)	42(80.8)/10 (19.2)	67(68.4)/31 (31.6)	0.105
Diabetes history (N/Y)	48 (92.3)/4 (7.7)	86 (87.8)/12 (12.2)	0.390
Smoking history (N/Y)	21 (40.4)/31 (59.6)	29 (29.6)/69 (70.4)	0.182
Drinking history (N/Y)	18 (34.6)/34 (65.4)	26 (26.5)/72 (73.5)	0.301
cT stage (T2/T3/T4a)	18 (34.6)/32 (61.5)/2 (3.9)	8 (8.2)/66 (67.3)/24 (24.5)	<0.001
cN stage (N0/N1/N2/N3)	12(23.1)/35(67.3)/5(9.6)/0(0)	14(14.3)/54(55.1)/26(26.5)/4(4.1)	0.027
cTNM stage (II/III/IVa)	28 (53.8)/22 (42.3)/2 (3.9)	14 (14.3)/60 (61.2)/24 (24.5)	<0.001
NLR (≤2.935/>2.935)	31 (59.6)/21 (40.4)	40 (40.8)/58 (59.2)	0.028
PLR (≤150.3/>150.3)	33 (63.5)/19 (36.5)	51 (52.0)/47 (48.0)	0.180
LMR (>3.27/≤3.27)	39 (75.0)/13 (25.0)	50 (51.0)/48 (49.0)	0.004
PNI (>50.15/≤50.15)	29 (55.8)/23 (44.2)	41 (41.8)/57 (58.2)	0.104
SII (≤672/>672)	29 (55.8)/23 (44.2)	48 (49.0)/50 (51.0)	0.428

LA-ESCC: locally advanced esophageal squamous cell carcinoma; pCR: pathological complete response; ECOG-PS: eastern cooperative oncology group performance status; BMI: body mass index; Y/N: yes/no; NLR: neutrophil to lymphocyte ratio; PLR: platelet to lymphocyte ratio; LMR: lymphocyte to monocyte ratio; PNI: prognostic nutritional index; SII: systemic immune-inflammation index; TNM: tumor node metastasis; U/M/L: upper/middle/lower; W/M/P: well/moderate/poor.

**Table 3:** Logistic univariate and multivariate analyses of predictors for pCR in LA-ESCC

	**Univariate analyses**		**Multivariate analyses**	
	**OR (95% CI)**	***P*-value**	**OR (95% CI)**	***P*-value**
Age (years, >60/≤60)	1.425 (0.679–2.914)	0.332		
Sex (male/female)	0.495 (0.163–1.494)	0.212		
ECOG-PS (1/0)	0.978 (0.441–2.170)	0.957		
BMI (kg/m^2^, >20/≤20)	0.853 (0.435–1.673)	0.644		
Tumor location (L/M/U)	0.862 (0.496–1.499)	0.589		
Differentiation (P/M/W)	0.449 (0.269–0.748)	0.002	0.464 (0.259–0.830)	0.010
Hypertension history (Y/N)	0.515 (0.229–1.157)	0.108		
Diabetes history (Y/N)	0.597 (0.183–1.954)	0.394		
Smoking history (Y/N)	0.620 (0.307–1.254)	0.184		
Drinking history (Y/N)	0.682 (0.330–1.410)	0.302		
cT stage (T4a/T3/T2)	0.201 (0.096–0.419)	<0.001		
cN stage (N+/N0)	0.462 (0.268–0.797)	0.006		
cTNM stage (IVa/III/II)	0.193 (0.100–0.374)	<0.001	0.225 (0.115–0.441)	<0.001
NLR (>2.935/≤2.935)	0.467 (0.236–0.927)	0.029		
PLR (>150.3/≤150.3)	0.625 (0.313–1.245)	0.181		
LMR (≤3.27/>3.27)	0.347 (0.165–0.729)	0.005	0.309 (0.132–0.707)	0.007
PNI (≤50.15/>50.15)	0.570 (0.289–1.125)	0.105		
SII (>672/≤672)	0.761 (0.387–1.496)	0.429		

LA-ESCC: locally advanced esophageal squamous cell carcinoma; pCR: pathological complete response; ECOG-PS: eastern cooperative oncology group performance status; U/M/L: upper/middle/lower; W/M/P: well/moderate/poor; Y/N: yes/no; BMI: body mass index; TNM: tumor node metastasis; NLR: neutrophil lymphocyte ratio; PLR: platelet to lymphocyte ratio; LMR: lymphocyte to monocyte ratio; PNI: prognostic nutritional index; SII: systemic immune-inflammation index; OR: odds ratio; CI: confidence interval.

### Establishment of a nomogram to predict pCR

**Figure 4.**
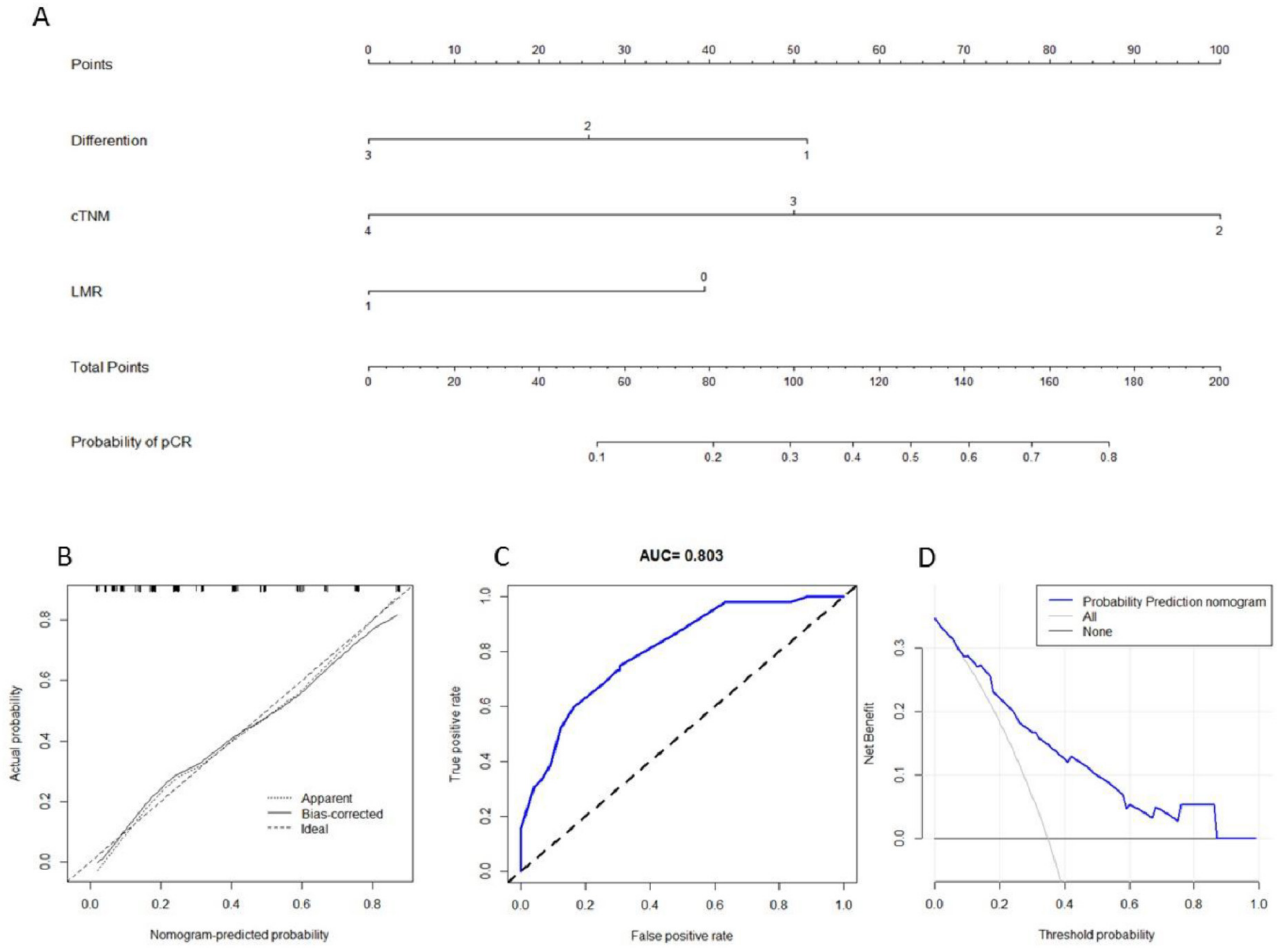
**Nomogram for pCR prediction.** (A) A predictive nomogram with the C-index of 0.779 including differentiation, cTNM, and LMR was established. Using a calibration plot with bootstrap sampling (*n* = 1000), the calibration revealed an acceptable agreement regarding pCR prediction internally (B); ROC (C), and DCA (D) indicated a good clinical applicability of the model in predicting pCR. cTNM; DCA: decision curve analysis; LMR: lymphocyte monocyte ratio; pCR: pathological complete response.

A predictive nomogram in LA-ESCC with the C-index of 0.779 including differentiation, cTNM, and LMR was established to predict pCR in nICT (Figure 4A). The calibration of the nomogram was carried out internally by bootstrap sampling (*n* = 1000), indicating that the model was well calibrated (Figure 4B). Good predictive ability and clinical applicability of the nomogram model for pCR prediction were found in ROC curve (AUC = 0.803) and DCA curve (Figure 4C-D).

## Discussion

We used several pretreatment clinical characteristics and hematological indexes to predict pCR. The study initially revealed that differentiation, cTNM, and LMR were independent predictors for pCR. Moreover, a nomogram model in LA-ESCC receiving nICT may accurately and effectively predict pCR. Our study focuses on the predictors from hematological indicators in predicting pCR before nICT. The results of this study will bring an important assessment of pCR using various pretreatment indexes before nICT in LA-ESCC patients.

Neoadjuvant treatment (nCT or nCRT) was recommended by the CSCO and NCCN guidelines [3, 4]. Immunotherapy has been a relatively modern innovation in the treatment of cancer in recent years. Based on the KEYNOTE and ATTRACTION studies, immunotherapy significantly improved the outcomes in patients with advanced ESCC. Therefore, immunotherapy was approved for first-line treatment for advanced ESCC [6, 7]. Following encouraging results in the advanced ESCC, nICT has gained much attention. Recently, nICT has had various promising outcomes in patients with LA-ESCC, such as high R0 resection rate, low adverse effects, high pCR rate, and limited postoperative complications [8–12]. However, most published studies focused on the efficiency and safety as well as pCR of nICT. Moreover, few studies have been conducted regarding predictors of pCR in LA-ESCC receiving nICT.

It is well known that patients with pCR after neoadjuvant therapy have a significantly long time of survival. Therefore, the prediction of pCR after neoagjuvant treatment has been a research hotspot in recent years. A variety of studies on ESCC reported that NLR, LMR, and PLR were correlated with pCR and prognosis [14–17]. A study including 87 cases of LA-ESCC reported that LMR was significantly higher in pCR patients compared to non-pCR patients and confirmed as an independent predictor in nCRT [14]. Another study demonstrated that pre-nCRT NLR and post-nCRT PLR were associated with pCR in 306 ESCC patients with nCRT [15]. Moreover, similar results were also found in PLR and SII in 311 ESCC patients who received nCRT [16]. These evidence indicate that peripheral blood parameters may have a certain significance for pCR prediction in ESCC with nCRT.

Recently, a study including 64 cases of LA-ESCC explored the relations between several hematological indicators and pCR in nICT [18]. However, the study was in a small sample, and the authors only focused on the pCR prediction between baseline and post-treatment indexes. Another study established a nomogram to predict TRG, but not for pCR, in 79 cases of LA-ESCC [19]. The authors developed a TRG prediction model and revealed that the changes in albumin and pretreatment white blood cells were significantly related to TRG. However, these two studies have some limitations. Firstly, the sample sizes of these two studies were small. Secondly, the predictors for pCR in nICT are still unclear. Thirdly, these two studies analyzed the blood indexes at baseline and after nICT treatment, but ignored the possibility of nICT itself might have some influence on these indicators after nICT. Fourthly, the above two studies included different immune checkpoint inhibitors that could influence the results. Our study included only one immune checkpoint inhibitor (camrelizumab) and initially revealed that LMR was an independent predictor of pCR. Results from our study provided new insights into nICT for LA-ESCC.

The exact mechanism between LMR and pCR remains unknown. There are some hypotheses on this issue. Firstly, lymphocyte can inhibit tumor cell proliferation and migration by inducing tumor cell apoptosis, which plays an important role in tumor immune surveillance and defense [26, 27]. Secondly, monocytes, especially tumor-associated macrophages, can reshape extracellular matrix, inhibit specific antitumor immunity and promote tumor proliferation, angiogenesis, progression, and metastasis by generating a series of cytokines [28, 29]. These reasons may explain why patients with higher LMR have higher pCR rates.

Our study developed an integrative nomogram model to predict pCR. Recent studies have reported that nomogram was a good method to predict factors in various cancers [15, 30–32]. Ajani et al. [30] and Toxopeus et al. [31] conducted two nomograms to predict pCR after nCRT in ESCC. They indicated that clinical data analyzed using a logistic regression model had a high probability of pCR prediction. The same results were also found in our study. The nomogram based on LMR, differentiation, and cTNM stage in LA-ESCC had good discrimination performance and calibration coordination for pCR prediction in nICT. Our study allows clinicians to use the model in their daily work to predict individual pCR in LA-ESCC before nICT.

Limitations should be acknowledged. Firstly, this was a retrospective study, which required attention to bias in data selection and collection. Secondly, the current study was a single-center study. Thirdly, hematological indexes may be influenced by various other factors. Fourthly, the nomogram model lacks external validation. Fifthly, the follow-up time for the current study was too short. Therefore, there was a lack of recurrence prediction. Finally, the basic biological and mechanisms regarding hematological indicators have not been thoroughly elucidated. Although limitations existed, our model may accurately and effectively predict pCR in LA-ESCC patients receiving nICT. Results from our study provided new insights for patients with LA-ESCC receiving nICT.

## Conclusion

Pretreatment LMR, differentiation, and cTNM stage are the predictors of pCR. Our study on nICT in LA-ESCC is of great significance for the current treatment. Nomogram based on LMR, differentiation, and cTNM stage in LA-ESCC may accurately and effectively predict pCR.

## Acknowledgments

The authors thank all the patients and their families who participated in this study.

**Conflicts of interest:** The authors declare no conflicts of interest.

**Funding:** The study was supported by grants from Zhejiang Medical and Health Science and Technology Project (2017KY237, 2018KY022). The study was also supported by Zhejiang TCM Science and Technology Project (2020ZB036, 2021ZB034 and 2022ZB051).
